# On speaking terms: a Delphi study on shared decision-making in maternity care

**DOI:** 10.1186/1471-2393-14-223

**Published:** 2014-07-09

**Authors:** Marianne J Nieuwenhuijze, Irene Korstjens, Ank de Jonge, Raymond de Vries, Antoine Lagro-Janssen

**Affiliations:** 1 Research Centre for Midwifery Science, Faculty Midwifery Education & Studies, Zuyd University, Universiteitssingel 60, 6229 ER Maastricht, the Netherlands; 2 Midwifery Science/EMGO Institute for Health and Care Research, VU University Medical Center, Van der Boechorststraat 7, 1081 BT Amsterdam, the Netherlands; 3 CAPHRI, University Maastricht, Universiteitssingel 60, 6229 ER Maastricht, the Netherlands; 4 Department of General Practice, Women Studies Medicine, Radboud University Medical Center, Geert Grooteplein-Zuid 10, 6525 GA Nijmegen, the Netherlands

**Keywords:** Choice, Shared decision-making, Maternity care, Delphi study, Pregnancy, Childbirth

## Abstract

**Background:**

For most women, participation in decision-making during maternity care has a positive impact on their childbirth experiences. Shared decision-making (SDM) is widely advocated as a way to support people in their healthcare choices. The aim of this study was to identify quality criteria and professional competencies for applying shared decision-making in maternity care. We focused on decision-making in everyday maternity care practice for healthy women.

**Methods:**

An international three-round web-based Delphi study was conducted. The Delphi panel included international experts in SDM and in maternity care: mostly midwives, and additionally obstetricians, educators, researchers, policy makers and representatives of care users. Round 1 contained open-ended questions to explore relevant ingredients for SDM in maternity care and to identify the competencies needed for this. In rounds 2 and 3, experts rated statements on quality criteria and competencies on a 1 to 7 Likert-scale. A priori, positive consensus was defined as 70% or more of the experts scoring ≥6 (70% panel agreement).

**Results:**

Consensus was reached on 45 quality criteria statements and 4 competency statements. SDM in maternity care is a dynamic process that starts in antenatal care and ends after birth. Experts agreed that the regular visits during pregnancy offer opportunities to build a relationship, anticipate situations and revisit complex decisions. Professionals need to prepare women antenatally for unexpected, urgent decisions in birth and revisit these decisions postnatally. Open and respectful communication between women and care professionals is essential; information needs to be accurate, evidence-based and understandable to women. Experts were divided about the contribution of professional advice in shared decision-making and about the partner’s role.

**Conclusions:**

SDM in maternity care is a dynamic process that takes into consideration women’s individual needs and the context of the pregnancy or birth. The identified ingredients for good quality SDM will help practitioners to apply SDM in practice and educators to prepare (future) professionals for SDM, contributing to women’s positive birth experience and satisfaction with care.

## Background

Women’s participation in decision-making is a growing expectation in maternity care. Women want to be involved in the decisions made during this important period of their lives [1-3], seeking to take responsibility for their own health and well-being as well as that of their baby [4]. Involvement in decision-making has a positive effect on their birth experiences and satisfaction with care [5-10]. Women look to their maternity care providers for support in making decisions. Care providers play a role in helping women to find, shift, and interpret information [4,11,12]. But as yet, maternity care providers themselves have little information about the best ways to share decision-making responsibilities with their clients, especially during labour and birth, when sharing decision-making can be more challenging.

Shared decision-making (SDM) is widely advocated as a way to support people in their healthcare choices [13-16]. SDM is defined as “an approach where clinician and patient share the best available evidence when faced with the task of making decisions, and where the patient is supported to consider options, to achieve informed preferences” [15]. SDM offers opportunities for greater mutual understanding through a dialogue between patient and care provider. The emphasis is on the process of coming to a decision. A number of authors have conceptualized SDM [17-19]. An extensive literature review identified a list of essential elements that must be present for patients and providers to engage in the process of SDM [17]. In the process of SDM the problem is explained and options are presented. Patient and care provider express their preferences, wishes and values, and together they explore beneficial solutions for the given situation. There is an interactive exchange of *professional information* (evidence on and experience with options, benefits, harms and uncertainties), *personal information* (circumstances and quality of life issues), *deliberation* by both parties based on disclosure of values and preferences for the particular situation, and *building towards a consensus-based decision* with shared responsibility. More recently, Elwyn (2012) presented a three step model for SDM in practice (Table 1) [18]: (1) choice talk, introducing the need for decision-making; (2) option talk, exploring the options and preferences; (3) decision talk, making the decision; and asked the experts to identify competencies necessary to perform these steps. Several authors have promoted a broad conceptualization of patients’ involvement in decision-making [20,21]. They emphasized the importance of the relationship between care provider and patient, where patients are enabled to consider their ‘best’ option, also taking into consideration individual circumstances from outside the clinical context and where patients can develop a positive sense of involvement.

**Table 1:** **Three-step model for SDM in clinical practice [**[18]**]**

	
Step 1.	**Choice talk**, introducing that a decision-making needs to be made and exploring what role the woman wants to play.
Step 2.	**Option talk**, exploring the woman’s values and preferences, informing her about the options and its consequences, deliberating with her and involving her partner or significant others.
Step 3.	**Decision talk**, making the final decision, safeguarding the woman’s sense of autonomy, clarity over the decision and informing other professionals involved in the care for the woman.

Maternity care providers can support and advise pregnant women in the many decisions they face during pregnancy, birth and postnatal; enabling women to take charge of their own choices in deliberation with their provider. Professional acceptance of SDM is still developing in maternity care [22,23]. Only recently, systematic reviews reported on decision aids to support women in their choices during pregnancy and birth [24-26]. Although these are important tools to enable evidence-based decision-making, these tools mainly focus on the *information* component of SDM. When making decisions around childbirth, there is more to consider than giving information about the available options. Birth is more than a physical experience, it is a family event influenced by cultural context and beliefs [27-29] and has a large emotional and social impact [5,30-32]. Decisions in the perinatal period often affect the physical, social, and psychological well-being of mothers and their babies.

Professional skills are essential for achieving SDM [33]. Some interesting work on competencies for SDM in medicine has been done, suggesting that more clarity on competencies is needed [34]. For maternity care, professionals need a clear picture of what contributes to good quality SDM during the perinatal period and what competencies are necessary to support women’s involvement in decision-making. These decisions may comprise choices between equal options that are – based on available evidence – comparable in effect, harms and benefits. But the process of SDM in maternity care is also relevant when options are not equivalent, and medically preferred options intervene with women’s preferences or beliefs. A careful process of deliberation and exchange can prevent escalation.

Research on SDM in medicine offers insight into the process of SDM in the consultation room [35], but research on SDM in the perinatal period is sparse. This is especially true for SDM in the dynamic process of labour and birth, where time can be limited by the need to make quick, on the spot decisions, and where the pain of the contractions and the need of the woman to stay focused on the birthing process may interfere with interaction and deliberation.

The aim of our study is to gain insight into the process of SDM during maternity care, first to identify and find consensus on ingredients for quality criteria for SDM in different situations during pregnancy and birth, and second to find consensus on professional competencies needed for SDM in maternity care.

## Methods

Between September 2012 and June 2013, we conducted a Delphi study. The Delphi method is widely used in health research to gain more understanding and/or consensus about a topic by anonymously bringing together and synthesizing the knowledge of geographically scattered experts [36,37]. A Delphi study consists of series of questionnaires or ‘rounds’ which are sent to experts to gather information. The definition of ‘expert’ in this method is related to theoretical knowledge, as well as knowledge from experience.

The research ethics committee of Atrium-Orbis-Zuyd assessed the project and confirmed that ethical approval was not needed (11 September 2012, number 12-N-107).

### Expert panel

The focus of our study was primarily on decision-making in everyday practice for healthy women and on the care providers that are mostly involved in the care for these women. In the Netherlands, like in other countries (e.g.: United Kingdom, New Zealand and Scandinavian countries), these women are cared for by midwives.

For our Delphi panel, we invited 71 experts who were active in the fields of SDM (8) and/or maternity care (63), including international opinion leaders. Email invitations were sent by the research team, describing the aim and design of the study and stating clearly the voluntary nature of the study. The experts were authors of key articles on SDM in general or on decision-making in maternity care, practitioners supervising pregnancies and births in different maternity care settings, researchers, educators, policy makers and representatives of care users. Because of the focus of our study, we invited a disproportionate number of experts from midwifery. The experts were from Europe (Cypress, Finland, Germany, Italy, Netherlands, Switzerland, UK), North-America (Canada, USA) and Australia; their disciplines included sociology, general medicine, obstetrics, midwifery, nursing, research and medical education.

### Design and data collection

The Delphi study had three iterative rounds; communication was in Dutch and English. All the experts were invited to participate via an email informing them of the purpose of the study, the process to be used and the estimated time it would take. Experts were asked for their willingness to participate in all rounds of the Delphi study. We explained that responses were confidential and that participation would be taken as informed consent. A subsequent email was sent to the experts who agreed to participate, containing a hyperlink to the Delphi website where the online Delphi questionnaire could be accessed using a password.

The study team used the responses of the first questionnaire to develop statements on 1) quality criteria for the process of SDM in different situations during the perinatal period and 2) competencies needed for SDM in maternity care. Subsequent emails with hyperlinks to the questionnaires of Round 2 and 3 were sent to the same pool of experts. In all rounds, non-responders received two reminders by email.

#### Round 1

Round 1 was exploratory, with the goal of revealing relevant components for the SDM process in different situations during the perinatal period and identifying the competencies needed for this. We used a questionnaire with open-ended questions. First, we asked the experts to describe their initial thoughts on SDM in maternity care and subsequently, we asked them how they would go about the communication process in order to come to shared decisions in different situations. We introduced Elwyn’s three-step model for SDM in clinical practice (Table 1) [18].

We used this information to develop a questionnaire with statements on quality criteria and competencies for SDM that was then distributed in Round 2 of the Delphi.

#### Round 2

The goal of Round 2 was to establish consensus about the importance of the statements for good quality of SDM in maternity care. The questionnaire listed 90 statements on quality criteria and competencies, introduced through exemplary cases from maternity care practice. The criteria were phrased in terms of observational behaviour of the care provider. The experts were asked to rate all statements [on a scale ranging from 1 to 7] for their significance for the SDM process in maternity care. Experts were also invited to elaborate on the statements or to suggest additional statements. Before we initiated Round 3, experts were informed of their own individual response to each statement, and the median score and range of the group in Round 2.

#### Round 3

In Round 3 we aimed to achieve final consensus on those statements where consensus had not been reached. The questionnaire included statements that were retained, modified or redeveloped from the Round 2 responses. Round 3 also allowed experts to edit and comment on the statements.

### Data analysis

Responses to the Round 1 questionnaire were grouped to identify recurring themes across experts’ responses. We analysed the responses from the user representatives separately to make sure that these were considered. A content analysis framework was used based on Elwyn’s three-step model for SDM [18]. Emerging and recurring themes were discussed with all authors and transcribed into statements on quality criteria for the SDM process and competencies needed for SDM in maternity care.

We used 7-point Likert scales ranging from ‘strongly disagree’ (1) to ‘strongly agree’ (7) to quantify and compare agreement on the statements in Rounds 2 and 3. A priori [37], we defined positive consensus as 70% or more of the experts scoring ≥6 (70% panel agreement), less than 5% scoring ≤3 (disagree) and a mean score of ≥6 with a standard deviation (SD) of ≤1.1. Negative consensus was defined as 70% or more of the experts scoring ≤2 (70% panel agreement), less than 5% scoring ≥5 (agree), and a mean score of ≤2 with a standard deviation (SD) of ≤1.1. Each round was analysed separately. Median scores (and range) were calculated to report back to the experts [37]. SPSS version 19.0 was used for the quantitative analyses.

## Results

We invited 71 experts (36 midwives, 19 obstetricians, 8 SDM experts, 8 representatives of users), 52 agreed to participate. Eight experts replied they could not participate because of “lack of time”, the other 11 experts did not respond. In Round 1, 48 experts filled out the questionnaire; 42 (88%) completed Round 2, and 32 of these 42 (76%) completed Round 3. Their characteristics are presented in Table 2.

**Table 2:** Socio-demographic characteristics of the experts

	**First round**	**Second round**	**Third round**
	**N = 48**	**N = 42**	**N = 32**
	**No. (%)**	**No. (%)**	**No. (%)**
Age (mean (SD))	45 (9.4)	45 (9.4)	45 (9.2)
Gender			
Female	43 (89.6)	39 (92.9)	30 (93.8)
Male	5 (10.4)	3 (7.1)	2 (6.3)
Background			
Midwife	31 (64.6)	29 (69.0)	24 (75.0)
Obstetrician	9 (18.8)	6 (14.3)	5 (15.6)
Physician	3 (6.3)	3 (7.1)	1 (3.1)
Representatives of care users	3 (6.3)	2 (4.8)	1 (3.1)
Other	2 (4.2)	2 (4.8)	1 (3.1)
Present professional activity*			
Maternity care	28 (58.3)	26 (61.9)	22 (68.8)
Research	15 (31.3)	11 (26.2)	9 (28.1)
Education	11 (22.9)	10 (23.8)	6 (18.8)
Professional organisation	5 (10.4)	5 (11.9)	4 (12.5)
Policy making	7 (14.6)	4 (9.5)	2 (6.3)
Work experience in years (mean (SD))			
Maternity care	12.5 (9.0)	12.0 (9.0)	12.7 (9.0)
Region in which currently active			
Netherlands	32 (66.7)	27 (64.3)	22 (68.8)
Europe	8 (16.7)	9 (21.4)	6 (18.8)
North America	7 (14.6)	5 (11.9)	3 (9.4)
Australia	1 (2.1)	1 (2.4)	1 (3.1)

*More than one activity is possible.

### Round 1

In Round 1, the experts expressed their views on SDM, offered suggestions for the woman-care provider interaction around decision-making and gave detailed input for quality criteria and competencies essential for SDM in different situations during the perinatal period. The main themes identified were: the woman-care provider relationship, care providers’ attitude and communication skills, enabling women to participate, exploration of preferences, women’s autonomy, information exchange, use of evidence, involvement of partners, tension around decision-making and decision-making when options are not equivalent or in urgent situations. The users in our panel specifically emphasized: being listened to, recognition of autonomy and involvement of the partner.

The overall response was that SDM is vitally important for women’s well-being and contributes to satisfying relationships between women and care providers. Several members of our expert panel emphasized “having enough time” and a “trusting woman-provider relationship” as essential conditions for SDM. The experts mentioned that the regular antenatal visits offer opportunities to build a relationship, anticipate situations that may occur and revisit complex issues. These visits also offer opportunities for providers to explore women’s values and expectations for the upcoming birth, allowing decisions during birth to be facilitated by an understanding fostered previously. Preparing women for an (urgent) decision in birth and discussing choices and preferences were identified as important aspects of antenatal care. Additionally, the experts expressed that providers need to be well-informed and up-to-date on findings from research, able to interpret evidence and apply it to the individual woman. Providers need to adjust their communication to the woman’s language when explain evidence. Translating complicated issues, such as risk, in understandable terms for women and their partners was seen as a challenge.

### Round 2

Using the responses of round 1, we identified 86 statements about quality criteria and 4 statements about competencies to include in the Round 2 questionnaire. We linked 48 out of the 86 statements to four exemplary decision-making scenarios that occur relatively frequent in maternity care. The other 38 quality criteria statements were focused on specific scenarios where disagreement between the woman and care provider about the preferred option influences the decision-making process (e.g. the wish for induction of labour); these will be reported in a separate article. The 4 competency statements were relevant for all the scenarios.

The four scenarios were:

•Decision-making scenarios during pregnancy:

I. decision with equal options (24 statements) and

II. decision with a clearly better option (7 statements);

•Decision-making scenarios during birth:

III. decision with equal options (11 statements) and

IV. urgent decision with a clearly better option (6 statements).

**Figure 1:**
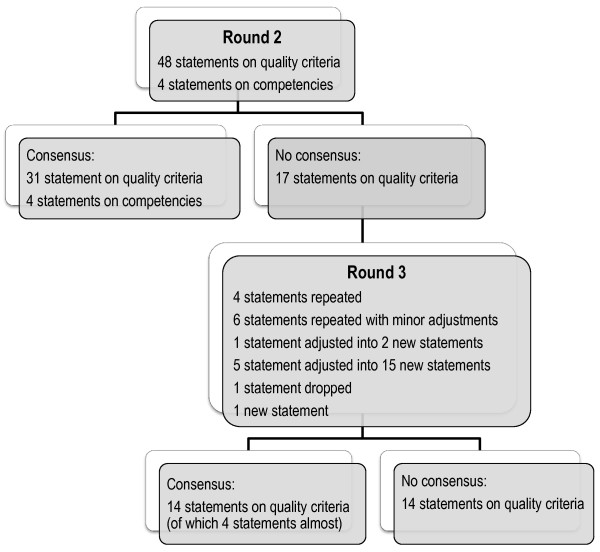
Consensus/non-consensus on statements in round 2 and 3.

Statements on scenario I, decisions during pregnancy with equal options, illustrate the basic process of SDM in maternity care. The quality criteria statements for this scenario were ordered according to Elwyn’s three-step model for SDM [18]: choice talk (5 statements), option talk (14 statements), and decision talk (5 statements). For scenarios II, III and IV, relevant quality criteria statements were added for each scenario. Additional file 1 presents all the statements and scores from rounds 2 and 3.In Round 2, consensus was reached on 35 (67%) of the 52 statements (Figure 1). Experts agreed on 31 quality criteria statements. These statements phrased the importance of a respectful dialogue, exploring the role women want to play in the decision-making process, encouraging her to play an active role, exploring her values and preferences, giving women accurate and accessible information and time to process and revisit this information, and making sure women’s autonomy is respected. When the options for decision-making are not equivalent, the experts agreed that it is still important to consider the woman’s thoughts and opinions.

There was also consensus on the need for care providers to be able to apply evidence, guidelines and decision aids to each woman’s individual situation.

When time for decision-making was limited during birth, the most important criteria were: preparing women antenatally for the possibility of quick decisions during birth, calmness of the care provider, briefly explaining the situation, seeking the woman’s consent and discussing the situation again after birth.

Consensus was reached on all four competency statements, with a level of agreement between 98% and 100%, and with mean scores between 6.6 and 6.7 (SD 0.49 to 0.73).

#### No consensus

No consensus was reached on 17 quality criteria statements.

There were two topics that showed a wide range in experts’ responses: input of the care provider’s advice and involvement of the partner in decision-making. We decided to explore these two topics further in Round 3, adjusting the 5 earlier quality criteria statements on these topics to 15 new statements to get better clarity where experts agreed.The comments of the experts in Round 2 led to minor adjustments in 6 other statements for Round 3. We also adjusted 1 statement into 2 new ones, dropped 1 statement and added 1 new statement. In total, the Round 3 questionnaire contained 28 quality criteria statements for decision-making in pregnancy and birth (Figure 1).

### Round 3

In this round, consensus was achieved on 10 of the 28 statements (36%), which led to a total of consensus in this Delphi study on 45 quality criteria statements and 4 competency statements (Figure 1). Another 4 statements nearly reached consensus with more than 70% (72 to 82%) of the experts scoring ≥ 6, but mean scores just below 6.

Experts agreed that for a good quality of SDM it is important that *communication*: should comprise an open dialogue with respect and empathy, and that care providers use understandable language, make clear agreements, are prepared to discuss decisions several times and make sure that other care providers are informed about the woman’s decisions. The *information* should be complete, evidence-based, and adjusted to women’s knowledge. Care providers should *support* women to be actively involved, identify their preferences and underlying motives, take time to process and revisit decisions, and respect women’s autonomy.

In case of no equivalent options for decision-making, listening, explaining and checking women’s understanding are important for *communication. Supporting* women by allowing them to explain their viewpoints, giving them accurate *information* and explicitly obtaining their consent were also seen as contributing to good quality decision-making in these situations.

During birth, experts agreed that it was important for good *communication* to be calm and to take time to explain even if those explanations must be brief in acute situations. Experts acknowledged that it was especially important to discuss the situation again after the birth (100% consensus; mean 6.9, SD 0.35). To *support* women’s involvement in decision-making during birth, experts agreed that preparations should start during antenatal care, making women aware that unforeseen decisions can occur and that time for decision-making may be limited, and that women’s expectations and preferences should be explored. During birth, providers should still seek women’s consent.

#### No consensus

After two rounds, no consensus was reached on the statements for the introduction of the “choice talk” [18] or on encouraging women to seek information from sources other than the care provider (agree: 55%; disagree: 10%). Also the experts did not reach full agreement on the statement *“Evidence-based guidelines are in principal the basis for decision-making”* (agree 69%, disagree 6%; mean 5.8 SD 1.24).

#### Care providers’ advice

In the further exploration of the topic on the input of care providers’ advice, the experts agreed on the statements *“The care provider makes sure that her/his preference is not forced upon the woman”* and *“The care provider puts forward her/his viewpoint based on evidence about the benefits and harms”*. Another statement nearly reached negative consensus: “*The care provider will never give her/his advice*” (disagree 69% (≤2), agree 3% (≥5); mean 2.3 SD 1.20). Experts did not reach agreement on care providers putting forward professional experience in their advice and were mainly negative about providers putting forward personal experience or their own preferences.

#### Involvement of the partner

Regarding the involvement of the partner in the decision-making process, experts agreed that partners should be involved in communication around information and deliberation of care options, but they did not reach consensus on involving partners in the final decision (59% agree, 3% disagree; mean 5.6, SD 1.01), or on the partner making the decision when the woman is unable to respond during birth (under the condition that the woman has consented) (53% agree, 3% disagree; mean 5.4, SD 1.08).

We ended the Delphi after the third round because saturation for consensus seemed to be reached.

## Discussion

A three-round Delphi study was conducted to identify quality criteria and professional competencies for SDM in maternity care and to explore the level of consensus among experts. Consensus was reached on 45 quality criteria statements and 4 competency statements (Table 3).

**Table 3:** Statements on quality criteria and competencies that reached consensus

	**Scenario**
**I**	**Interaction around decisions during PREGNANCY**
	Decisions with more or less equal (treatment) options or decisions with inconclusive evidence that one option is better than the others.
	***Choice talk***
	The care provider creates an open dialogue to discuss the choices and decisions based on respect, empathy, trust and comfort.
	The care provider explores which role the woman is willing to play in the decision-making process.
	The care provider encourages all women to play an active role in the decision-making process and supports her throughout.
	***Option talk***
	The care provider is aware of the available evidence, guidelines and decision aids, is capable of assessing their quality, and can apply them to the woman’s individual situation.
	The care provider explores what the woman already knows and provides additional or corrective information if necessary.
	The care provider provides objective and accurate information on the available options.
	The care provider informs the woman using accessible language tailored to her social and cultural background.
	The care provider explores available options, also those the woman is not immediately interested in.
	The care provider explores the values and preferences of the woman.
	The care provider explores the underlying motives for the woman’s preferences.
	The care provider gives the woman ample time and space to process this information.
	Complex decisions are discussed over the course of several consultations.
	With the woman's consent, the care provider will involve the partner in the decision-making process.
	The care provider involves the partner in the conversation around information.
	The care provider involves the partner in the deliberation of the options.
	The care provider respects the woman’s choice to involve a third party in the decision-making process.
	The woman should always feel autonomy in the decision-making process.
	***Decision talk***
	Once a decision is taken, it is clearly stated.
	The care provider verifies whether the decision was understood.
	The care provider stresses that the woman can change her mind about her decision at any time.
	During the pregnancy, the care provider revisits the decisions that were made.
	The care provider will inform other care providers involved in the care for the woman about the woman's decisions and underlying motivations with.
	The care provider makes sure that the autonomy of the woman is respected
	The care provider makes sure that her/his preference is not forced upon the woman.
	The care provider puts forward her/his viewpoint based on evidence about the benefits and harms.
**II.**	**Interaction around decisions during PREGNANCY**
	Decisions with an option that is clearly better - based on research or experience.
	If there is an option that is clearly better, the care provider will explain this to the woman.
	The care provider encourages the woman to express her thoughts and opinions.
	The care provider listens to and respects the woman's input.
	The care provider ensures that the woman has understood the information provided.
	If the woman is responsive, the care provider will always ask for informed consent.
**III.**	**Interaction around decisions during BIRTH**
	Decisions with more or less equal (treatment) options or decisions with inconclusive evidence that one (treatment) option is better than the others.
	During the pregnancy, the care provider discusses the possibility of unforeseen decision moments during birth.
	During the pregnancy, the care provider explores with the woman possible dilemmas surrounding decisions during birth.
	During the pregnancy, the care provider discusses the woman's needs, preferences and expectations concerning labour and birth, and puts the preferences on paper (e.g. in a birth plan).
	The care provider makes it clear that the woman can change her mind about any decisions and choices regarding her birth plan.
	Preferably, a woman in labour should not be confronted with choices or decisions for the first time.
	The care provider exudes calm and takes the time to explain and discuss the situation.
	The care provider briefly describes the essence of the situation and the available options.
	The care provider always checks whether the woman has heard and understood her/him.
	The woman will always be asked for her consent.
**IV.**	**Interaction around decisions during BIRTH**
	Urgent decisions with an option that is clearly better - based on research or experience.
	During the pregnancy, the care provider explains that acute situations may arise during birth that require quick decisions.
	The care provider takes a moment to explain the situation to the woman and her partner.
	The care provider strives to eliminate a rushed feeling.
	During an acute situation, the care provider explains that s/he will take the lead.
	If possible, the care provider obtains the explicit consent of the woman before taking any measures.
	The care provider will discuss the situation again after the birth.
**V.**	**Competencies**
	Establish a relationship and open dialogue with the woman (and her partner) based on respect and recognition of cultural diversity.
	Evaluate available evidence and experience, and provide the woman with accurate, honest information in the context of her individual situation.
	Enable and activate the woman to participate in the decision-making process, support her to deliberate about the options and express her preferences and views.
	Reduces tension and guides the process to reach a shared decision.

SDM in maternity care was seen as a dynamic process that starts in antenatal care and ends after birth. Experts agreed that the regular visits during pregnancy offer opportunities to build a relationship, anticipate situations that may occur and revisit complex decisions. Professionals should prepare women antenatally for unexpected, urgent decisions in birth and discuss these decisions again with women postnatally. Open and respectful communication between women and care professionals is essential; information needs to be accurate, evidence-based and understandable to women. The experts saw establishing a relationship with the woman as an important professional competency for shared decision-making.

Experts were divided about the contribution of professional advice in shared decision-making and about the partner’s role. They agreed that care professionals can put forward their viewpoints based on evidence, but did not find consensus on putting forward viewpoints based on professional or personal experience. They also agreed that the partner should be involved when giving information and deliberating the options, but did not find consensus on the involvement of the partner in the final decision.

### Strengths and weaknesses

In this study we explored a topic that is very relevant for everyday maternity practice and so far has received little attention in research. The results offer midwives and other maternity care providers suggestions for applying SDM in the context of birth. They are also relevant for other health care situations were urgent decision-making in a limited time frame can be anticipated or occurs.

Strength of the study is the use of a Delphi consensus process. Boulkedid [36] confirms that a Delphi is very appropriate for identifying quality criteria for health care and we applied their recommendations for planning, using, and reporting the Delhi procedure. Experts of a Delphi on quality of care should reflect the full range of stakeholders [36]. Diverse stakeholders often have different points of view about quality of care [38], which may enrich the results. Our international expert panel included health professionals (midwives and obstetricians), representatives of users and SDM methodologists. A potential weakness is the skewed expert demographics. Because the focus of our study was primarily on decision-making in everyday practice for healthy women, the majority of the experts were Dutch midwives. However, we kept a critical cut-off level by requiring less than 5% scoring of ≤3 (disagree) before accepting consensus, thus guaranteeing that if more than two experts disagreed with a statement, it would not be accepted. Only a few user representatives engaged in the study. It is possible that unfamiliarity with the Delphi technique played a role in their willingness to participate. Their responses to the open-ended questions of Round 1 were of high value for the development of the statements for rounds 2 and 3, but our findings need to be validated in larger groups of users. The fact that the experts were all from high-income countries should be considered when applying the quality criteria and competencies in care for women from other cultural backgrounds [39,40].

From the invited experts, 32% did not participate in the Delphi, mainly because of lack of time. Despite our information beforehand explaining the Delphi procedure, two reminder e-mails in each round, and feedback after Round 2, nearly one-third of the participants dropped-out. A Delphi study is a long process which makes it harder for participants to make a full commitment; the numbers of drop-outs are comparable with other Delphi studies [41-43].

We only asked the participants to rate the criteria on “importance” of the statement for the quality of decision-making. Preferably, factors such as feasibility are also considered. However, the questionnaire was extensive and we were sensitive to the burden placed on the experts.

### General results in context

Several studies describe key elements of SDM [17,18,43,44]. Our study found similar key elements for SDM in maternity care: open dialogue, stimulating women to participate in decision-making, interactive exchange of accurate information tailored to women’s individual understanding, and giving women sufficient time to consider options.

Additionally, we identified new elements with specific importance for SDM in maternity care. Many decisions in maternity care are made outside the consultation room, when women are labouring and time is limited. Nevertheless, women want to participate in decision-making during birth [2,5]. Specific quality criteria were identified for SDM during birth, including situations with urgent decision-making. Full SDM is not always possible in these situations, but preparations during pregnancy, a trusting relationship, briefly explaining what is happening and discussing the decisions again after birth, will enhance women’s feeling of involvement [9,10,45]. The preparation for SDM in birth can be integrated in the antenatal talks on preparing for birth and contain the elements of ‘choice talk’ and ‘decision talk’ without specifically going into every possible event at birth*.* Although evidence is limited, studies in other medical fields indicate that there is no evidence that SDM is not feasible in emergency situations [46].

SDM is sometimes presented as the choice between treatment options [35]. In maternity care, decisions are often about choosing between ‘watchful waiting’ and intervening to address a possible risk of adverse outcomes. These two options are sometimes hard to compare as the meaning of a relatively higher risk is open to individual interpretation, and certain interventions (e.g. a hospital birth) may have consequences for women’s preferences or existential view of life. SDM is highly relevant in these situations with early, respectful deliberation, clear explanation of different options, and encouragement for women to express their thoughts and opinions [47,48].

Others found that patients seemed to place more value on the process of involvement in sharing decisions than on who finally makes the decision [49]. Our findings also emphasize the importance of a focus on the process in SDM: a process that starts in pregnancy and ends after birth – when important decisions are revisited and discussed – and that aims for mutual understanding of preferences, values, and evidence.

### Specific results in context

Experts were hesitant about the contribution of care providers’ advice in SDM. Although, they almost unanimously disagreed with the statement ‘*care providers will never give advice*’, implying that there is a role for care providers’ advice, they seemed reluctant to exert a strong influence on women’s choices and see providers’ primary role as supporting women to make their own choices. The literature on SDM indicates that care providers can introduce their own opinions and experiences, when done in an unthreatening way [35]. Given the many events in pregnancy and childbirth and an overwhelming amount of information, women often ask care providers for advice. This underscores SDM in maternity care as a dynamic process, in which providers need to balance between supportive and directive approaches suited to the context and the needs of the woman [50]. In some circumstances, e.g. choices around prenatal screening, the emphasis is on supporting women to make their own choice, while on other occasions, e.g. in emergencies, a more directive approach – based on antenatal discussions – may be necessary.

Experts in our panel were also hesitant to give the partner a full part in the making of the decision. They agreed that the partner should be involved when giving information and deliberating the options, but felt that the final decision-making lies with the woman. There is a legal base for this and experts’ cautiousness may be based on the vulnerability of some women in the relationship with their partner. However, it is possible that women, recognizing that they can be withdrawn into themselves during birth, may have agreed beforehand that their partner will be their advocate for the decisions that must be made. In Round 1, the user representatives frequently emphasized the involvement of their partners in every aspect of decision-making. It is important to recall that the perinatal period is a transition to parenthood for the partner as well. The partner should feel involved and recognized as there is a responsibility for the child from the minute it is born and mutual involvement is a strong base for the start of a good family life [51,52]. Care providers have the difficult task to assess each time whether partner’s involvement benefits the woman, and try to act accordingly.

The fact that women are involved in decision-making gives them a share of the responsibility for the choices and the outcomes. Several experts in our panel remarked that this could be a burden to women, especially if the outcome is disappointing. Skilful providers offer support, but it may not always be easy to identify when support is needed, leading to patients’ perception of ‘abandonment’ [48]. Women and their partners should also be made aware that not everything in pregnancy and birth can be controlled, unexpected things may happen. Even though the responsibility is shared, this does not mean that care providers are less responsible. Discourses of equality in responsibility can hide the fact that the health professional has legal obligations in the event of a poor outcome [53].

### Further needs for research

Our study is only one of the steps towards full understanding and use of SDM in maternity care. Next, the results of this Delphi have to be brought back to a comprehensive set of quality criteria, which need to be validated in larger groups of care users and different maternity care professionals. Additional research is needed to explore the feasibility and performance of the quality indicators in everyday practice and to identify interventions, education programmes and implementation strategies that can support users and professionals in the application of SDM in practice. Given the changes taking place in maternity care, of special interest would be to look at interprofessional collaboration around SDM and at the decision-making process in group consultations [54,55].

## Conclusion

SDM in maternity care is a dynamic process taking into consideration women’s individual needs and the context of the pregnancy or birth. The identified ingredients for good quality SDM will help practitioners to apply SDM in practice and educators to prepare (future) professionals for SDM. Supporting women in the many decisions they face during the perinatal period will contribute to a positive birth experience and satisfaction with care.

Based on our results, we recommend an active and committed role of the professional, and a decision-making process that is tailored to the needs, circumstances, and capacities of women. This process should be characterized by openness, a willingness to explore options, and mutual respect.

## Competing interests

The authors declare that no conflicts of interests are related to this study.

## Authors’ contribution

All authors conceived and designed the study. MN acquired the data and carried out the text analysis. The responses and themes were read independently by IK. MN carried out the other and analysis drafted the manuscript. All authors interpreted the results, critically revised the manuscript for important intellectual content, and contributed to and approved the final version. TLJ supervised the project. MN and IK had full access to all of the data in the study and take responsibility for the integrity of the data and the accuracy of the data analysis.

## Pre-publication history

The pre-publication history for this paper can be accessed here:

http://www.biomedcentral.com/1471-2393/14/223/prepub

## Supplementary Material

Additional file 1 Results of the Delphi Shared decision-making in maternity care, round 2 and 3. Click here for file
