# Supplementary material for: On speaking terms: a Delphi study on shared decision-making in maternity care
Source: BMC Pregnancy Childbirth. 2014 Jul 9;14:223. doi: 10.1186/1471-2393-14-223 (PMC4104734; doi:10.1186/1471-2393-14-223)
Supplement: Additional file 1 — Results of the Delphi Shared decision-making in maternity care, round 2 and 3. [file 1471-2393-14-223-S1.docx]

**On speaking terms: A Delphi study on shared decision-making in maternity care**

Marianne J Nieuwenhuijze RM MPH, Irene Korstjens PhD, Ank de Jonge RM PhD, Raymond de Vries PhD, Toine Lagro-Janssen MD PhD

**Additional file 1**

**Results of the Delphi Shared decision-making in maternity care, round 2 and 3**

Four decision-making scenarios in maternity care were used as introduction to the statements in Delphi round 2 and 3.

| **I. Interaction around decisions during PREGNANCY**  Decisions with more or less equal (treatment) options or decisions with inconclusive evidence that one option is better than the others. | **Scenario I**  Ms A (gravida 3, para 2; 35 weeks pregnant) and her care provider discuss the plans for her mode of birth, assuming a full term birth. Ms A had a physiological birth the first time and a caesarean section the second time because of a breech presentation. The options and all accompanying pros and cons are discussed. |
| --- | --- |
| **II. Interaction around decisions during PREGNANCY**  Decisions with an option that is clearly better - based on research or experience. | **Scenario II**  Ms B (gravida 1, para 0) is 42 weeks pregnant. She is diagnosed with an elevated blood pressure. Further treatment is discussed and the care provider suggests induction of labour. |
| **III. Interaction around decisions during BIRTH**  Decisions with more or less equal (treatment) options or decisions with inconclusive evidence that one option is better than the others. | **Scenario III**  Ms C (gravida 2, para 1) is in labour and progressing nicely. The contractions are painful and the care provider notices that the woman is uncomfortable in her current position. The care provider mentions switching to a different birthing position. |
| **IV. Interaction around decisions during BIRTH**  Urgent decisions with an option that is clearly better - based on research or experience. | **Scenario IV**  Ms D (gravida 1, para 0; 40 weeks pregnant) is giving birth in the hospital. She has been pushing for 30 minutes after a normal first stage of labour. The foetal heart sounds indicate foetal distress and the care provider suggests an assisted delivery. |

|  |  | *% agree = % of experts scoring ≥ 6 on 7-point Likert-scale  †% disagree = % of experts scoring ≤ 3 on 7-point Likert-scale | | | |
| --- | --- | --- | --- | --- | --- |
| I. Interaction around decisions during PREGNANCY with more or less equal options |  | **Results** | | | |
| **round 2** | **round 3** | **round 2** | | **round 3** | |
| ***Choice talk*** |  |  | |  | |
| 1. At the start of the discussion, the care provider clearly indicates the need for a decision. | 1. During a conversation on such a topic, the care provider clearly indicates that in the end a decision needs be made. | % agree*  % disagree†  mean (SD) | 45.2  2.4  4.57 (2.09) | % agree  % disagree  mean (SD) | 56.3  3.1  5.41 (1.34) |
| 2. At the start of the discussion, the care provider clearly indicates the timeframe in which a decision must be made. | 2. During the conversation, the care provider clearly indicates that there is a timeframe in which a decision needs to be made (not necessarily during this conversation). | % agree  % disagree  mean (SD) | 45.2  26.2  4.88 (1.93) | % agree  % disagree  mean (SD) | 62.5  6.3  5.56 (1.01) |
| 3. The care provider creates an open dialogue to discuss the choices and decisions based on respect, empathy, trust and comfort. |  | % agree  % disagree  mean (SD) | 92.9  0%  6.57 (0.70) |  |  |
| 4. The care provider explores which role the woman is willing to play in the decision-making process. |  | % agree  % disagree  mean (SD) | 78.6  2.4  6.10 (1.03) |  |  |
| 5. The care provider encourages all women to play an active role in the decision-making process and supports her throughout. |  | % agree  % disagree  mean (SD) | 78.6  2.4  6.21 (1.03) |  |  |
| ***Option talk*** |  |  |  |  |  |
| 6. The care provider is aware of the available evidence, guidelines and decision aids, is capable of assessing their quality, and can apply them to the woman’s individual situation. |  | % agree  % disagree  mean (SD) | 92.9  2.4  6.48 (0.92) |  |  |
| 7. The care provider explores what the woman already knows and provides additional or corrective information if necessary. |  | % agree  % disagree  mean (SD) | 88.1  2.4  6.17 (1.01) |  |  |
| 8. The care provider provides objective and accurate information on the available options. |  | % agree  % disagree  mean (SD) | 95.2  2.4  6.52 (0.89) |  |  |
| 9. The care provider informs the woman using accessible language tailored to her social and cultural background. |  | % agree  % disagree  mean (SD) | 100  0  6.69 (0.47) |  |  |
| 10. The care provider explores available options, also those the woman is not immediately interested in. | 10. The care provider explores available options, also those the woman is not immediately interested in. | % agree  % disagree  mean (SD) | 73.8  2.4  5.88 (0.97) | % agree  % disagree  mean (SD) | 81.5  3.1%  5.88 (0.83) |
| 11. The care provider explores the values and preferences of the woman. |  | % agree  % disagree  mean (SD) | 90.4  0  6.45 (0.74) |  |  |
| 12. The care provider brings forward her/his own experiences with the different options. | Replaced by statements e., f. and g. | % agree  % disagree  mean (SD) | 14.3  33.3  4.02 (1.46) |  |  |
| 13. The care provider will only offer her/his advice and underlying motivations at the woman's request. | Offered as m., complemented by statements i., j., k., and l. | % agree  % disagree  mean (SD) | 21.4  42.9  3.95 (1.74) |  |  |
| 14. The care provider gives the woman ample time and space to process this information. |  | % agree  % disagree  mean (SD) | 92.9  0  6.50 (0.63) |  |  |
| 15. Complex decisions are discussed over the course of several consultations. |  | % agree  % disagree  mean (SD) | 92.9  2.4  6.36 (0.79) |  |  |
| 16. The care provider encourages the woman to obtain information from different sources (friends/family, consulting other experts or the Internet, etc.) and discuss this with her/him. | 15. The care provider encourages the woman to obtain information from different sources (friends/family, consulting other experts or the Internet, etc.) and discuss this with him/her. | % agree  % disagree  mean (SD) | 54.8  9.5  5.33 (1.39) | % agree  % disagree  mean (SD) | 65.6  6.3  5.38 (1.16) |
| 17. With the woman's consent, the care provider will involve the partner in the decision-making process. |  | % agree  % disagree  mean (SD) | 78.6  4.8  6.00 (1.06) |  |  |
| 18. The care provider respects the woman’s choice to involve a third party in the decision-making process. |  | % agree  % disagree  mean (SD) | 81  0  6.17 (0.91) |  |  |
| 19. The woman should always feel autonomy in the decision-making process. |  | % agree  % disagree  mean (SD) | 85.7  0  6.33 (0.90) |  |  |
| ***Decision talk*** |  |  |  |  |  |
| 20. Once a decision is taken, it is clearly stated. |  | % agree  % disagree  mean (SD) | 92.9  0  6.45 (0.71) |  |  |
| 21. The care provider verifies whether the decision was understood. |  | % agree  % disagree  mean (SD) | 95.2  0  6.64 (0.57) |  |  |
| 22. The care provider stresses that the woman can change her mind about her decision at any time. |  | % agree  % disagree  mean (SD) | 85.7  0  6.33 (0.90) |  |  |
| 23. During the pregnancy, the care provider revisits the decisions that were made. | 22. During the pregnancy, the care provider revisits the decisions that were made. | % agree  % disagree  mean (SD) | 69  2.4  5.86 (1.00) | % agree  % disagree  mean (SD) | 78.1  3.1%  6.00 (1.02) |
| 24. The care provider will inform other care providers involved in the care for the woman about the woman's decisions and underlying motivations with. |  | % agree  % disagree  mean (SD) | 81  2.4  6.29 (1.02) |  |  |
|  | a. (new): The care provider explores the underlying motives for the woman's preferences. |  |  | % agree  % disagree  mean (SD) | 84.4  0  6.31 (0.74) |

| II. Interaction around decisions during PREGNANCY with an option that is clearly better |  | **Results** | | | |
| --- | --- | --- | --- | --- | --- |
| **round 2** | **round 3** | **round 2** | | **round 3** | |
| 25. If there is an option that is clearly better, the care provider will explain this to the woman. |  | % agree  % disagree  mean (SD) | 92.9  2.4  6.36 (0.96) |  |  |
| 26. If a better option exists, the care provider will use information to direct the woman to this option. | Based on comments from the experts this statement was deleted in round 3. | % agree  % disagree  mean (SD) | 40.5  23.8  4.83 (1.67) |  |  |
| 27. The care provider encourages the woman to express her thoughts and opinions. |  | % agree  % disagree  mean (SD) | 100  0  6.71 (0.46) |  |  |
| 28. The care provider listens to and respects the woman's input. |  | % agree  % disagree  mean (SD) | 95.2  0  6.64 (0.58) |  |  |
| 29. The care provider ensures that the woman has understood the information provided. |  | % agree  % disagree  mean (SD) | 97.6  0  6.76 (0.48) |  |  |
| 30. If the woman is responsive, the care provider will always ask for informed consent. | 30. If the woman is responsive, the care provider will always ask for informed consent. | % agree  % disagree  mean (SD) | 76.2  4.8  6.12 (1.25) | % agree  % disagree  mean (SD) | 93.8  3.1%  6.63 (0.91) |
| 31. The involvement of the woman in the decision-making process will take place within the medical frameworks set by the care provider. | 31. Evidence-based guidelines are in principal the basis for decision-making. | % agree  % disagree  mean (SD) | 47.6  19  4.88 (1.77) | % agree  % disagree  mean (SD) | 68.8  6.3  5.78 (1.24) |

| III. Interaction around decisions during BIRTH with more or less equal options |  | **Results** | | | |
| --- | --- | --- | --- | --- | --- |
| **round 2** | **round 3** | **round 2** | | **round 3** | |
| 32. During the pregnancy, the care provider discusses the most common decision-making points during active labour. | 32a. During the pregnancy, the care provider discusses the possibility of unforeseen decision moments during birth. | % agree  % disagree  mean (SD) | 71.4  7.1  5.81 (1.29) | % agree  % disagree  mean (SD) | 75  0  5.84 (0.77) |
|  | 32b. During the pregancy, the care provider explores with the woman possible dilemmas surrounding decisions during birth. |  |  | % agree  % disagree  mean (SD) | 71.9  3.1%  5.81 (0.90) |
| 33. During the pregnancy, the care provider discusses the woman's needs, preferences and expectations concerning labour and birth, and puts the preferences on paper (e.g. in a birth plan). |  | % agree  % disagree  mean (SD) | 90.5  0  6.43 (0.67 |  |  |
| 34. The care provider makes it clear that the woman can change her mind about any decisions and choices regarding her birth plan. |  | % agree  % disagree  mean (SD) | 90.5  0  6.50 (0.74) |  |  |
| 35. The woman should not be confronted with choices or decisions for the first time during active labour. | 35. Preferably, a woman in labour should not be confronted with choices or decisions for the first time. | % agree  % disagree  mean (SD) | 73.8  4.8  5.90 (1.25) | % agree  % disagree  mean (SD) | 81.3  0  6.25 (0.76) |
| 36. The care provider exudes calm and takes the time to explain and discuss the situation. |  | % agree  % disagree  mean (SD) | 97.6  0  6.69 (0.60) |  |  |
| 37. The care provider briefly describes the essence of the situation and the available options. |  | % agree  % disagree  mean (SD) | 92.9  4.8  6.19 (1.11) |  |  |
| 38. The care provider always checks whether the woman has heard and understood her/him. |  | % agree  % disagree  mean (SD) | 95.2  0  6.50 (0.59) |  |  |
| 39. The care provider will explain her/his preference at the woman's request. | Replaced by statements d. and h. | % agree  % disagree  mean (SD) | 45.2  16.7  5.02 (1.47) |  |  |
| 40. The care provider will inform the partner (and/or third party) and involve them in the decision-making process. | Replaced by statements n., o. and p. | % agree  % disagree  mean (SD) | 50  7.1  5.40 (1.47) |  |  |
| 41. Only if the woman is unresponsive, decisions can be made in consultation with the partner. | Replaced by statement q. | % agree  % disagree  mean (SD) | 50  9.5  5.29 (1.44) |  |  |
| 42. The woman will always be asked for her consent. |  | % agree  % disagree  mean (SD) | 83.3  2.4  6.38 (0.96) |  |  |

| IV. Interaction around decisions during BIRTH with urgent decisions and an option that is clearly better |  | **Results** | | | |
| --- | --- | --- | --- | --- | --- |
| **round 2** | **round 3** | **round 2** | | **round 3** | |
| 43. During the pregnancy, the care provider explains that acute situations may arise during birth that require quick decisions. |  | % agree  % disagree  mean (SD) | 76.2  0  6.12 (0.99) |  |  |
| 44. The care provider takes a moment to explain the situation to the woman and her partner. |  | % agree  % disagree  mean (SD) | 90.5  2.4  6.48 (0.99) |  |  |
| 45. The care provider strives to eliminate a rushed feeling. |  | % agree  % disagree  mean (SD) | 88.1  4.8  6.21 (1.09) |  |  |
| 46. During an acute situation, the care provider explains that s/he takes charge. | 46. During an acute situation, the care provider explains that s/he will take the lead. | % agree  % disagree  mean (SD) | 64.3  19  5.38 (1.77) | % agree  % disagree  mean (SD) | 71.9  9.4  5.59 (1.34) |
| 47. The care provider obtains the explicit consent of the client before taking any measures. | 47. If possible, the care provider obtains the explicit consent of the woman before taking any measures. | % agree  % disagree  mean (SD) | 52.4  14.3  5.21 (1.73) | % agree  % disagree  mean (SD) | 84.4  3.1  6.25 (1.11) |
| 48. The care provider will discuss the situation again after the birth. |  | % agree  % disagree  mean (SD) | 100  0  6.86 (0.35) |  |  |

| V. Competencies |  | **Results** | |
| --- | --- | --- | --- |
| **round 2** |  | **round 2** | |
| 1. Establish a relationship and open dialogue with the woman (and her partner) based on respect and recognition of cultural diversity. |  | % agree  % disagree  mean (SD) | 97.6  0  6.67 (0.53) |
| 2. Evaluate available evidence and experience, and provide the woman with accurate, honest information in the context of her individual situation. |  | % agree  % disagree  mean (SD) | 100  0  6.64 (0.49) |
| 3. Enable and activate the woman to participate in the decision-making process, support her to deliberate about the options and express her preferences and views. |  | % agree  % disagree  mean (SD) | 97.6  0  6.64 (0.53) |
| 4. Reduces tension and guides the process to reach a shared decision. |  | % agree  % disagree  mean (SD) | 97.6  2.4  6.60 (0.73) |

| VI. Input of the care provider in the process of shared decision-making. |  | **Results** | |  |
| --- | --- | --- | --- | --- |
|  | **round 3** | **round 3** | |  |
|  | The care provider |  | |  |
|  | c. … makes sure that the autonomy of the woman is respected | % agree  % disagree  mean (SD) | 96.9  0  6.56 (0.56) |  |
|  | d. … makes sure that her/his preference is not forced upon the woman. | % agree  % disagree  mean (SD) | 90.6  0  6.41 (0.76) |  |
|  | e. … puts forward her/his viewpoint based on evidence about the benefits and harms. | % agree  % disagree  mean (SD) | 90.6  3.1  6.25 (0.84) |  |
|  | f. … puts forward her/his viewpoint based on professional experience. | % agree  % disagree  mean (SD) | 56.3  3.1  5.50 (1.05) |  |
|  | g. … puts forward her/his viewpoint based on personal experience. | % agree  % disagree  mean (SD) | 12.5  46.9  3.75 (1.46) | % disagree (≤2) = 21.9 |
|  | h. … puts forward her/his viewpoint based on her/his own preference. | % agree  % disagree  mean (SD) | 3.1  84.4  2.69 (1.26) | % disagree (≤2) = 46.9 |
|  | i. … puts forward her/his viewpoint after assessment of the woman's situation. | % agree  % disagree  mean (SD) | 34.4  19.7  4.88 (1.39) |  |
|  | j. … only puts forward a viewpoint at the woman's request. | % agree  % disagree  mean (SD) | 21.9  34.4  4.19 (1.53) |  |
|  | k. … always offers her/his advice and underlying motivations. | % agree  % disagree  mean (SD) | 53.1  15.6  5.03 (1.75) |  |
|  | l. … will never offer her/his advice. | % agree  % disagree  mean (SD) | 3.1  81.2  2.31 (1.20) | % disagree (≤2) = 68.7  % agree (≥5) = 3.1% |
|  | m. … will only offer her/his advice and underlying motivations at the woman's request. | % agree  % disagree  mean (SD) | 12.5  37.5  4.00 (1.59) |  |

| VII. Involvement of the partner in the process of shared decision-making. |  | **Results** | |  |
| --- | --- | --- | --- | --- |
|  | **round 3** | **round 3** | |  |
|  | After it is clear that the woman agrees, … |  | |  |
|  | n. … the care provider involves the partner in the conversation around information. | % agree  % disagree  mean (SD) | 84.4  0  6.22 (0.87) |  |
|  | o. … the care provider involves the partner in the deliberation of the options. | % agree  % disagree  mean (SD) | 75  0  5.97 (0.86) |  |
|  | p. … the care provider involves the partner in the decision. | % agree  % disagree  mean (SD) | 59.4  3.1  5.63 (1.01) |  |
|  | q. Only when - during birth - the woman is unable to respond can decisions be made in consultation with the partner (on the condition that it is clear that the woman has consented to this). | % agree  % disagree  mean (SD) | 53.1  3.1  5.44 (1.08) |  |
